# Comparative analysis of transcriptional changes in zebrafish *cep290* and *bbs2* mutants by RNA-seq reveals upregulation of inflammatory and stress-related pathways

**DOI:** 10.3389/fnmol.2023.1148840

**Published:** 2023-05-24

**Authors:** Sarah E. Grabinski, Dhwani Parsana, Brian D. Perkins

**Affiliations:** ^1^Department of Ophthalmic Research, Cole Eye Institute, Cleveland Clinic, Cleveland, OH, United States; ^2^Department of Ophthalmology, Cleveland Clinic Lerner College of Medicine, Case Western Reserve University, Cleveland, OH, United States; ^3^Department of Molecular Medicine, Cleveland Clinic Lerner College of Medicine, Case Western Reserve University, Cleveland, OH, United States

**Keywords:** *cep290*, regeneration, zebrafish, *bbs2*, Müller cell 22

## Abstract

Acute injury to the adult zebrafish retina triggers the release of pro-inflammatory cytokines and growth factors that stimulate multiple gene regulatory networks, which ultimately stimulate Müller glia to proliferate and regenerate neurons. In contrast, zebrafish carrying mutations in *cep290* or *bbs2* undergo progressive loss of cone photoreceptors and exhibit signs of microglia activation and inflammation, but the mutants fail to stimulate a regeneration response. To identify transcriptional changes that occur in zebrafish mutants undergoing progressive photoreceptor degeneration, RNA-seq transcriptional profiling was performed on *cep290*^−/−^ and *bbs2*^−/−^ retinas. The PANTHER Classification System was used to identify biological processes and signaling pathways that were differentially expressed between mutants and wild-type siblings during degeneration. As expected, genes associated with phototransduction were downregulated in *cep290* and *bbs2* mutants compared to wild-type siblings. Although both *cep290* and *bbs2* mutants undergo proliferation of rod precursors in response to retinal degeneration, the process of negatively regulating proliferation is enriched for upregulated genes, and this negative regulation may restrict proliferation of Müller glia and inhibit regeneration. A total of 815 differentially expressed genes (DEGs) were shared by *cep290* and *bbs2* retinas. Genes in pathways associated with inflammation, apoptosis, stress response, and PDGF signaling were overrepresented. Identifying the genes and biological pathways that are common in zebrafish models of inherited retinal degeneration provides a foundation for future studies on the mechanisms that regulate cell death as well as processes that prohibit Müller cell reprogramming or proliferation in a model capable of retinal regeneration. The pathways will provide targets for future interventions that may promote successful regeneration of lost photoreceptors.

## Introduction

Ciliopathies are a collection of rare genetic disorders that result from defects in the genesis or function of primary cilia (Reiter and Leroux, 2017). Cilia are microtubule-based structures that function like cellular antennae to detect extracellular stimuli and mediate intracellular signaling. Cilia are critically important for cellular physiology, developmental signaling, and organ function. Unsurprisingly, ciliopathies exhibit a broad range of overlapping clinical features and are best considered a spectrum of diseases with considerable clinical and genetic heterogeneity (Waters and Beales, 2011; Van De Weghe et al., 2022). Pathogenic disease variants in more than 190 genes have been linked to ciliopathies, with some genes associated with multiple ciliopathy disorders (Coppieters et al., 2010). Syndromic ciliopathies exhibit variable pleiotropy and include the lethal disease Meckel syndrome (MKS), Joubert Syndrome (JBTS), Bardet-Biedl Syndrome (BBS), and Senior-Løken Syndrome (SLS). In other cases, the defect is isolated to a single tissue and results in non-syndromic disease, such as retinitis pigmentosa (RP) or Leber Congenital Amaurosis (LCA).

Retinal degeneration is one of the most common clinical features of ciliopathies but other ocular phenotypes can occur. Retinopathy frequently presents with nystagmus that progresses to a rod-cone dystrophy but cases of cone-rod dystrophy have been reported (Riise et al., 2002; Mockel et al., 2011; Denniston et al., 2014). The onset, progression, and degree of vision loss varies across diseases and between patients diagnosed with the same disorder. JBTS results from mutations in more than 40 genes and patients present with consideration variation in ocular phenotypes. Nystagmus is the most common phenotype and retinal dystrophy and reduced electroretinograms (ERG) recordings were found in approximately 35% of patients (Wang et al., 2018). BBS is an autosomal recessive disorder caused by pathogenic variants in at least 21 genes (*BBS1-BBS21*) and more than 90% of BBS patients experience retinal degeneration by teens to early twenties (Denniston et al., 2014; Chandra et al., 2021). LCA is a severe non-syndromic retinal dystrophy that results from pathogenic variants in at least 25 genes. Mutations in the gene *CEP290* (*Centrosomal protein of 290 kDa*) are a leading cause of LCA in Caucasians. LCA is initially characterized by nystagmus and visual impairment at birth or in the first few years of life and functional vision rapidly deteriorates (Tsang and Sharma, 2018; Daich Varela et al., 2022).

The mutations in ciliopathy genes disrupt distinct components of the cilium, yet all cause retinal degeneration. In BBS, many mutations occur in genes that encode protein subunits of the BBSome, which is an octomeric complex that functions as an adaptor for protein transport within the cilium (Nachury et al., 2007; Wingfield et al., 2018; Ye et al., 2018; Chandra et al., 2021). In the absence of BBSome components, membrane proteins accumulate in the photoreceptor outer segment and are thought to contribute to degeneration. Knockout mouse models exist for several BBSome components and all undergo photoreceptor death, although with varied rates of degeneration (Chandra et al., 2021). The Cep290 protein is a component of the photoreceptor connecting cilium, which is analogous to the transition zone of primary cilia. The transition zone serves as a gate for entry of ciliary proteins. Mutations in *CEP290* can lead to BBS, JBTS, and LCA (Coppieters et al., 2010). The Cep290 protein decorates the length of the microtubules within the photoreceptor connecting cilium (Potter et al., 2021). However, the function of Cep290 is enigmatic and roles in cilia assembly, trafficking, and structural stability have all been proposed (Craige et al., 2010; Barbelanne et al., 2015; Li et al., 2016). While imaging and biochemical studies continue to clarify the perspectives on how these proteins function in photoreceptors, whether photoreceptor death in ciliopathies is triggered by similar cellular mechanisms remains unclear.

Assessing changes in gene transcription during photoreceptor degeneration can reveal those cellular pathways and processes that are common between different ciliopathies. In this study, we used bulk RNA-seq and bioinformatics to assess the transcriptomic changes in the retinas of the zebrafish *bbs2* and *cep290* mutants during the peak of photoreceptor death. Bbs2 is a component of the BBSome and the zebrafish *bbs2* mutant undergoes a progressive cone degeneration with a peak at approximately 4 months post fertilization (Song et al., 2020). The zebrafish *cep290* mutant also exhibits progressive cone degeneration and lacks approximately 50% of cones at 6 mpf (Lessieur et al., 2019; Fogerty et al., 2022). In zebrafish, the Müller glia cells exhibit a robust ability to regenerate lost neurons in response to injury (Goldman, 2014; Hyde and Reh, 2014), yet the *bbs2* and *cep290* mutants fail to regenerate. The purpose of this study was to identify differentially expressed genes (DEGs) and to determine the common cellular pathways that were altered in the retinas of both *bbs2* and *cep290* mutants. Disruption to these ciliary genes led to elevated inflammatory signaling, increased expression of solute transporters, and a reduction in visual response gene function. The common pathways suggest that death of photoreceptors triggers an inflammatory response that contributes to photoreceptor death in *bbs2* and *cep290* models.

## Methods

### Animal maintenance

Adult zebrafish were maintained and housed in an Aquatic Habitats recirculating system (Pentair) with a 14/10 h light/dark cycle with ambient room lighting. Zebrafish lines used in this study included the mutant lines *cep290*^fh297^ (Lessieur et al., 2019) and *bbs2* (Song et al., 2020). Animals from heterozygous crosses of *cep290* or *bbs2* lines were genotyped using high-resolution melt analysis (HRMA) as previously described (Lessieur et al., 2019; Song et al., 2020). Experiments included animals of both sexes. All animal procedures were done with approval by the Institutional Animal Care and Use Committee (IACUC) at the Cleveland Clinic.

### Dissection, RNA preparation, qRT-PCR, and sequencing

Fish at 6 months post-fertilization (mpf) of age (*n* = 4) were dark adapted overnight for 16–20 h and euthanized in the dark. Retinas were dissected from whole eyes with minimal inclusion of the RPE. Four independent samples, each of which contained two pooled retinas, were collected. RNA was purified using Trizol (15596026, Thermofisher), with Glycoblue (AM9515, Thermofisher) used as a co-precipitant as previously described (Fogerty et al., 2022). Samples were DNase treated (Turbo DNase, AM2238, ThermoFisher) and purified via lithium chloride precipitation. All samples had RNA Integrity Numbers ≥8.8. A total of 500 ng of purified RNA was used for reverse transcription. Reverse transcription was performed with the Bio-Rad iScript cDNA synthesis kit per instructions. Quantitative qPCR reactions were performed on 5 ng/uL cDNA using Bio-Rad SsoFast EvaGreen Supermix kit. Primer sequences are provided in Table 1. Three technical replicates were performed for each biological sample. Reactions were run on a Bio-Rad CFX96 Touch Real-Time PCR detection system and analyzed using CFX Manager. Fold changes were calculated by the ΔΔC_t_ method, with 18S rRNA used for normalization. A total of 500 ng of RNA from each sample was submitted for sequencing. Samples were depleted of ribosomal RNA during library preparation and sequenced on an Illumina NovaSeq 6000 using paired-end 150 bp (*cep290*) or 100 bp (*bbs2*) reads. All library preparations, quality control, and next-generation sequencing was performed by the Lerner Research Institute (LRI) Genomics Core at the Cleveland Clinic.

**Table 1:** Primer sequences for qRT-PCR.

Target	Forward sequence	Reverse sequence
*stat1a*	5′ AGTCGCAGCAATGACTCAGTG 3′	5′ CTGCTGATGATCATCGCCATTG 3′
*il-11a*	5′ CCGGTTCAAGTCTCTTCCAG 3′	5′ AGGTTTGCATGGAGCTGAGA 3′
*irf1b*	5′ TGAAATCATGCCCGTGTCCA 3′	5′ TACCTGTGTGAATGGCCCAC 3′
*irf9*	5′ AGGAGGCATCTATGCCAAGC 3′	5′ TGCGGAAACATTCCACTCTTG 3′
*irf3*	5′ AACAGCGACGATGTGCTC 3′	5′ ATCTGGGTTCCTGGATCC 3′
*atf3*	5′ TCACGCTGGACGACTTCACAAACT 3′	5′ TCTCAGTGTTCATGCAGGCTCTGT 3′
*atf5*	5′ TGGTGAACGCAAACAGAAGA 3′	5′ GCTGTTCCTCCAATGAGTCC 3′
*jun*	5′ CGCTTTCTCTCAGCATGACAGT 3′	5′ GATTGAGCGTCATGTTGTGTTTC 3′
*mych*	5′ CCCGACCGCTTAAAACTGGA 3′	5′ CTCATCGTCAAACAGCAACGG 3′
*mycl1b*	5′ GGTCAGAATCTCGCACCGACTC 3′	5′-ATGTGGAAACGCTTCATGCAGG 3′
*junbb*	5′ TACACGACGCTGAACGCATA 3′	5′ GTATGTGGGACGGCAGGTAG 3′
*cd44a*	5′ GAAAGTAATGCGAAGGAG 3′	5′ TCATCAGTGCCACAATCT 3′
*fosab*	5′ GTGAACGAAACAAGATGGCTG 3′	5′ TTTCATCCTCAAGCTGGTCAG 3′

### Differential gene expression

Reads were mapped to the genome (D.rerio-ENSEMBL-grcz11.r104: downloaded June 28, 2021) and gene abundance estimated using the star aligner v2.7.5a (Dobin et al., 2013). Transcripts with a *p-*value < 0.05 and a false discovery rate (FDR) < 0.05 were considered differentially expressed genes (DEGs). DESeq2 v1.30.1 (Love et al., 2014) was used to assess raw counts for differential expression and normalize raw counts between samples with a regularized log transformation. For genes that are highly expressed, the rlog values are approximately equivalent to log_2_ of the counts, while for genes with lower expression, the value approaches the average expression for that gene across all samples. Normalized counts of the total combined DEGs from *cep290* and *bbs2* (*n* = 5522) were used for the clustering analysis comparing *cep290* samples to *bbs2* samples to account for differences in sequencing depth between both samples and genes within each experiment. To adjust for the difference in total reads between the *cep290* and *bbs2* experiments, z-scores were calculated across the 8 samples (4 wild-type, 4 mutant) within each experiment. The mean count was set to 0 for each gene, with the sample score representing the number of standard deviations away from that mean. Log_2_ fold change was used for the clustering analysis to compare differential gene expression of both mutants to differential gene expression that occurs during the course of a 28-day retinal regeneration in zebrafish (NCBI Gene Expression Omnibus accession no: GSE180518; Kramer et al., 2021). The Euclidean distance between each gene for each sample was calculated, and samples were clustered using the unweighted pair group method with arithmetic mean (UPGMA) algorithm, which calculates sample distance based on the average difference across all genes.

### PANTHER gene ontology analysis

PANTHER version 17.0 (Mi and Thomas, 2009; Thomas et al., 2022) was used map genes to ontology terms using the Gene Ontology (GO) Biological Processes, GO-Slim Molecular Function, and PANTHER Pathways databases. There were 25,704 genes in the *Danio rerio* reference. In *cep290*, 16,873 were uniquely mapped to an ontology term, 116 had multiple mapping information, and 4818 were unmapped. In *bbs2*, 16,957 were uniquely mapped to an ontology term, 115 had multiple mapping information, and 4818 were unmapped. A binomial test was used to identify ontologies that were enriched for upregulated or downregulated DEGs. Ontologies were considered enriched if DEGs were two-fold or greater more common than expected with *p* < 0.05. The PANTHER over-representation test (Mi et al., 2019) was performed to identify ontologies whose genes were more abundant than expected among the most differentially expressed genes in common between *cep290* and *bbs2*, using Fisher’s Exact test with a FDR correction. The most differentially expressed genes were defined as DEGs where the absolute value of the log_2_ fold change (|log_2_ fold change|) was >0.5 or ~ 1.4-fold (*n* = 413). This was approximately the top 50% of DEGs in both experiments. Of these genes, 341 were uniquely mapped to the genome, 2 had multiple mapping information, and 72 were unmapped. Ontologies were considered overrepresented if they had a FDR < 0.05.

### Statistical analysis

DESeq2 analyses were performed using R v4.0.4. Python v3.9.13 was used to parse JSON results from PANTHER and perform the clustering analysis. The following packages were used: pandas v1.5.0, numpy v1.23.3 (Harris et al., 2020), statistics (D’Aprano, 2013), scipy v1.9.1 (Virtanen et al., 2020), json v2.0.9 (Ippolito, 2008), and seaborn v0.12.0 (Wickham et al., 2019; Waskom, 2021). The binomial test was performed and all other figures were generated using R v4.2.2. The following packages were used: tidyverse v1.3.2 (Wickham et al., 2019), janitor v2.1.0 (Firke, 2021), forcats v0.5.2 (Wickham, 2022), tidytext v0.3.4 (Silge and Robinson, 2016), ggtext v0.1.2 (Wilke and Wiernik, 2022), qdapRegex v0.7.5 (Rinker, 2022), scico v1.3.1 (Lin Pederson and Crameri, 2022).

### Data availability

*cep290* mapped reads and pairwise comparisons can be found in Supplementary Table 1. *bbs2* mapped reads and pairwise comparisons can be found in Supplementary Table 2.

## Results

**Figure 1:**
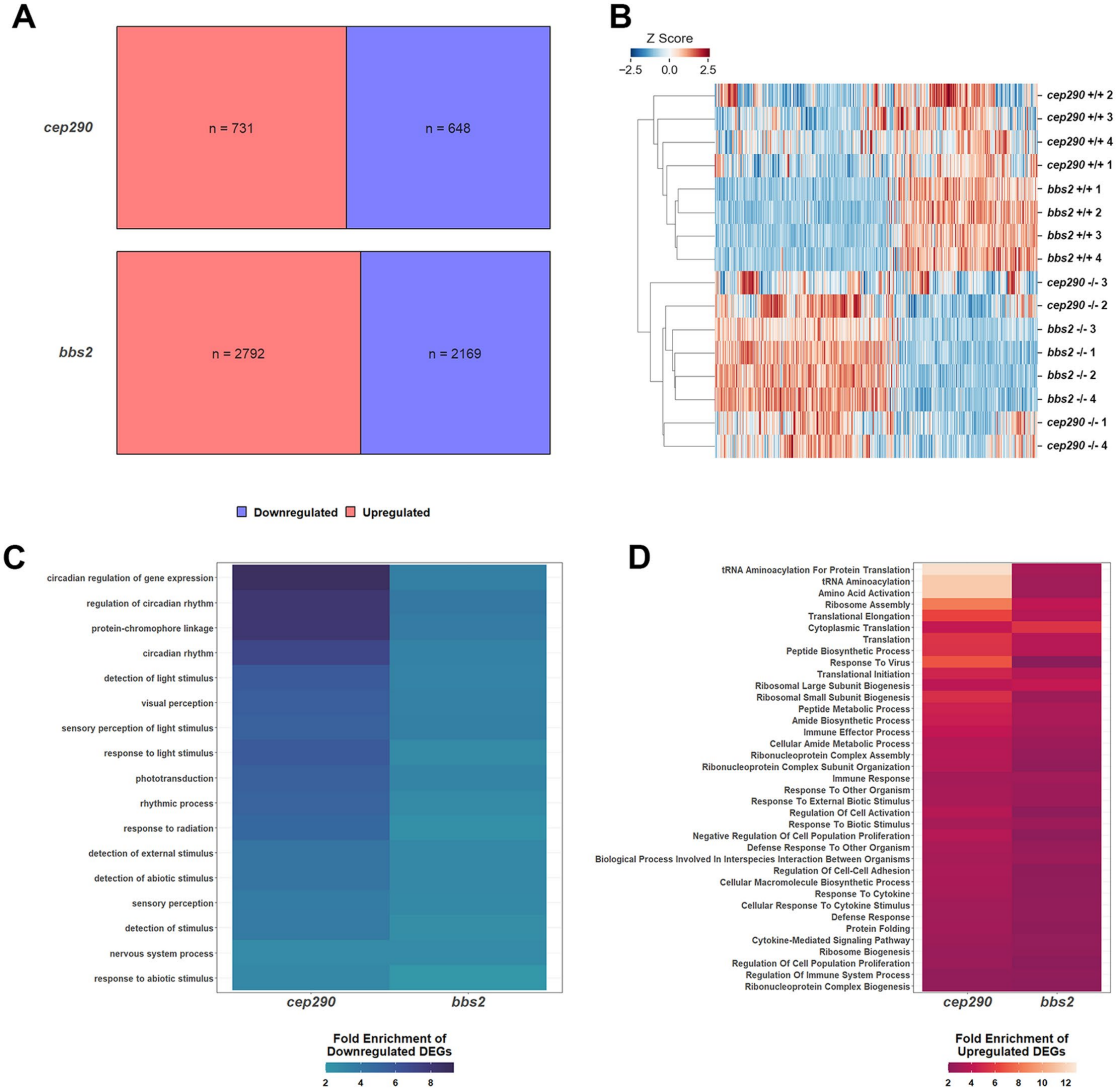
Differentially expressed genes (DEGs) in retinas of *cep290*^−/−^ and *bbs2*^−/−^ versus wild-type zebrafish regulate similar biological processes. **(A)** Upregulated (red) and downregulated (blue) DEGs in retinas from 6 mpf *bbs2*^−/−^ and *cep290*^−/−^ mutants compared to wild-type siblings. **(B)** Heat map showing the standardized gene counts of DEGs in all samples (*n* = 5,522) and hierarchically clustered by sample. The length of the branch is proportional to the difference in transcript levels between samples. **(C)** Heat map of all downregulated PANTHER Biological processes based on DEGs from *cep290* and *bbs2* mutant retinas. **(D)** Heat map of all upregulated PANTHER Biological processes based on DEGs from *cep290* and *bbs2* mutant retinas.

The phenotype of the *cep290* and *bbs2* zebrafish models of retinal ciliopathies is characterized by the degeneration of photoreceptors, chronic inflammation, and increased proliferation of rod precursor cells (Song et al., 2020; Fogerty et al., 2022). To identify core gene regulatory networks that underlie progressive photoreceptor degeneration in zebrafish, we performed bulk RNA-seq on retinas from *cep290* and *bbs2* mutants and wild-type siblings at 6 months post fertilization (mpf) when degeneration was ongoing. Total RNA was extracted and purified from dark-adapted retinas (*n* = 4 per genotype), which had the retinal pigment epithelium (RPE) removed. Individual libraries were prepared from each retina sample and all libraries subjected to next-generation sequencing. The average number of total reads was 1.12×10^8^ per *cep290* library and 3.00×10^7^ per *bbs2* library. Differentially expressed genes (DEGs) were defined as those genes with *p* < 0.05 and a false discovery rate (FDR) < 0.05. This definition does not rely on a minimal fold-change criteria, but rather identifies those transcripts expressed in *cep290* and *bbs2* retinas that exhibit a statistically detectable difference in expression from wild-type across all samples. The *cep290* dataset had 1379 DEGs, with 731 upregulated DEGs and 648 downregulated DEGs (Figure 1A). The *bbs2* dataset had 4961 DEGs with 2792 upregulated DEGs and 2169 downregulated DEGs.

To adjust for differences in read depth between the *cep290* and *bbs2* experiments, each sample within each dataset (n_*cep290* + wild-type_ = 8; n_*bbs2* + wild-type_ = 8) was given a z-score by gene equal to the number of standard deviations away from the mean count for that dataset across the 8 samples. A heat map illustrating hierarchical clustering of the datasets using all DEGs in both mutants (*n* = 5522) revealed a strong similarity in expression patterns between the *cep290* and *bbs2* mutant samples and that differed from the wild-type samples (Figure 1B). The datasets from the *cep290* and *bbs2* mutant samples were intermixed on a single major branch, illustrating the high degree of similarity between the two mutant transcriptomes.

To identify biological processes with differential gene expression, genes were mapped to ontology terms in the GO Biological Processes PANTHER database. The binomial test was used to identify processes with at least a two-fold enrichment of up- or downregulated DEGs over the proportion that would be expected at random. Consistent with ongoing degeneration of photoreceptors, several biological processes related to light perception were enriched for downregulated DEGs (Figure 1C). Also enriched for downregulated DEGs were circadian rhythm processes, possibly as this pathway contains DEGs that are also involved in visual response functions. Several pathways associated with immune system activation and cytokine signaling were enriched for upregulated DEGs in both *cep290* and *bbs2* (Figure 1D). This is consistent with elevated inflammatory signaling observed in both mutants. We also noticed that several pathways associated with protein biosynthesis were enriched for upregulated DEGs, suggesting that both mutants respond to retinal degeneration by increasing protein synthesis related to secretion of inflammatory molecules. The negative regulation of cell population proliferation pathway also contained more upregulated DEGs, suggesting that mechanisms that inhibit regeneration may be activated in chronic disease states.

**Figure 2:**
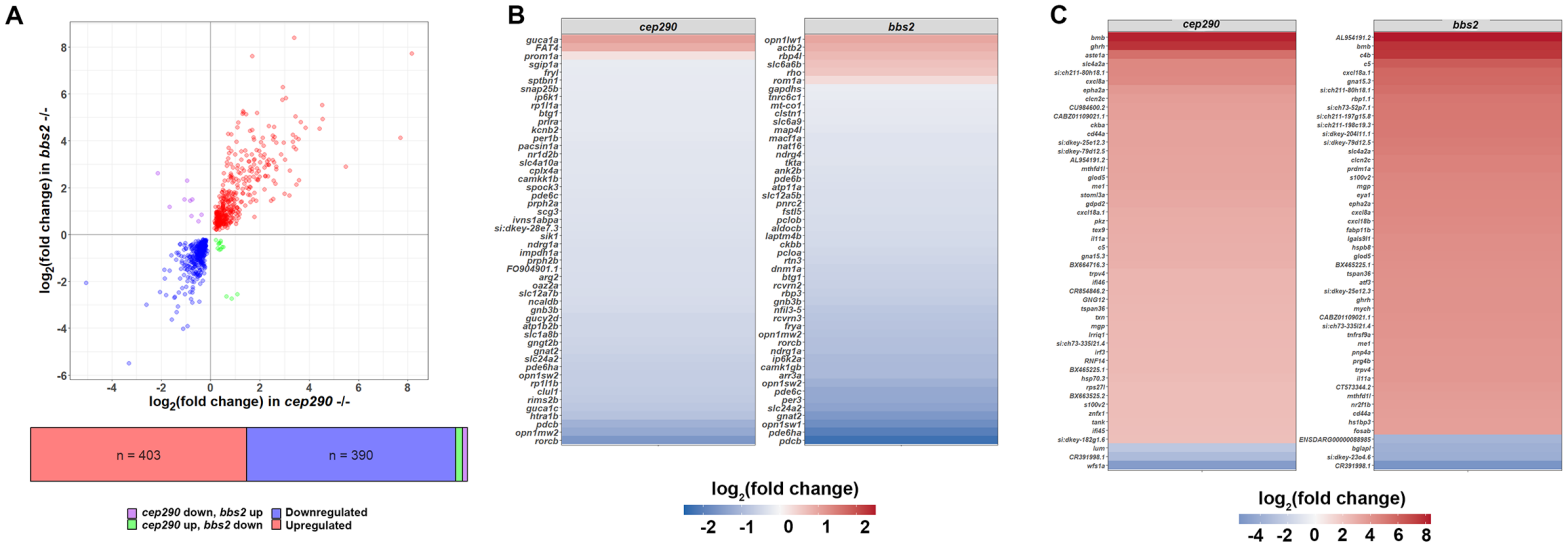
Enrichment of differentially expressed genes in biological processes of interest in *cep290* and *bbs2* mutants. **(A)** Log/log plot of DEG fold changes of common DEGs in *cep290^−/−^* and *bbs2^−/−^* datasets. Blue circles represent downregulated DEGs. Red circles represent common upregulated DEGs. Purple circles are DEGs upregulated in *bbs2^−/−^* retina but downregulated in *cep290^−/−^* retinas. Green circles are DEGs downregulated in *bbs2^−/−^* retinas but upregulated in *cep290^−/−^* retinas. **(B)** Heat map of the log_2_ fold change in the top 50 most abundantly expressed of the 815 DEGs in common by read count. Most DEGs were downregulated. **(C)** Heat map of the log_2_ fold change in the top 50 DEGs with greatest fold change in expression.

From these analyses, we anticipated that a common set of DEGs in the *cep290* and *bbs2* retinas would provide information on shared biological pathways that are altered during degeneration. A total of 815 DEGs were shared between the *cep290* and *bbs2* datasets, of which 403 (49.4%) were upregulated in both, 390 (47.9%) were downregulated in both, 13 (1.6%) were upregulated in *cep290* but downregulated in *bbs2*, and 9 (1.1%) were downregulated in *cep290* but upregulated in *bbs2* (Figure 2A). The log_2_ fold changes in the *cep290* and *bbs2* datasets were highly correlated (Pearson’s *r* = 0.838, *p* < 0.0001), indicating that photoreceptor degeneration in both models produced a common set of DEGs. The 50 DEGs found to be expressed most abundantly among these DEGs in common between the mutant retinas were also predominantly downregulated when compared to wild-type retinas (Figure 2B). Among these downregulated genes were several cone-specific genes, including opsins (*opn1sw2*, *opn1mw2*), the alpha subunit of cone transducin (*gnat2*), cone cGMP phosphodiesterase (*pde6c*), and the solute carrier gene *slc24a2*, which encodes an ortholog of the potassium-dependent sodium-calcium exchanger found in cone photoreceptors. The 50 DEGs with the greatest fold-change in expression were predominantly upregulated in both mutants (Figure 2C). Among these are the C-X-C motif chemokines *cxcl8a* and *cxcl18a.1*, which function to attract neutrophils and leukocytes to injured tissues, the cytokine *il11a*, which is orthologous to human IL-11, the cell adhesion molecule *cd44a*, which functions in apoptotic and autophagy pathways (Cao et al., 2022), and growth hormone releasing hormone *ghrh*, which is highly expressed in the developing retina (Krueckl et al., 2003).

**Figure 3:**
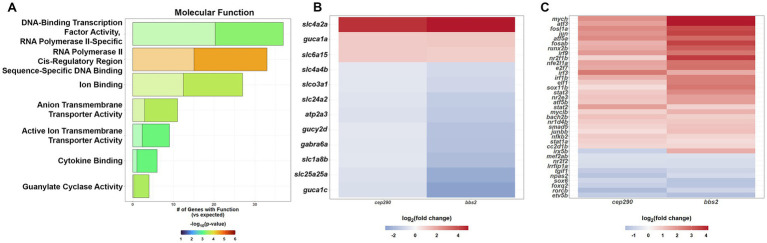
PANTHER Overrepresentation Analysis of the molecular function of the DEGs shared by *cep290* and *bbs2* mutants with a |log_2_ > 0.5| fold change in expression. **(A)** GO Molecular Function analysis to identify the molecular functions that were over- or underrepresented for the common DEGs. **(B)** Cone-specific genes expressed at lower levels in both mutants **(C)** PANTHER Pathway analysis to identify signaling pathways that are over- or underrepresented for common DEGS.

We next identified all DEGs that were common between *cep290* and *bbs2* and that also had at least a 1.4-fold (log_2_ > 0.5) change in expression (up- or downregulated; *n* = 413) and used the PANTHER GO-Slim Molecular Function enrichment analysis to identify specific classes of molecules playing mechanistic role within a pathway. This analysis revealed an overabundance of genes functioning in guanylate cyclase activity, active ion transmembrane transporter activity, anion transmembrane transporter activity, cytokine binding and transcription (Figure 3A). Guanylate cyclases and guanylate cyclase activating proteins (GCAPs) are enzymes responsible for the synthesis of the second messenger cGMP. Mammalian photoreceptors express two membrane-bound retinal guanylate cyclases (RetGC-1 and RetGC-2). RetGC-1 is more abundant and mutations in RetGC-1 lead to cone-rod dystrophy and Leber’s congenital amaurosis (Perrault et al., 1996; Duda et al., 1999). Zebrafish express three GCs (zGC1, zGC2, zGC3), with zGC1 and zGC2 expressed in rods and zGC3 expressed exclusively in cones (Ratscho et al., 2009; Collery and Kennedy, 2010). zGC3 is encoded by the *gucy2d* gene and by sequence homology is the ortholog to the human RetGC-1 (Ratscho et al., 2009). Three GCAPs are expressed in zebrafish (GCAP1, GCAP2, GCAP3), with GCAP1 and GCAP2 expressed in rods and GCAP3 expressed exclusively in cones (Imanishi et al., 2002). GCAP3 is encoded by the *guca1c* gene. Finally, the solute carrier gene *slc24a2* encodes an ortholog of the potassium-dependent sodium-calcium exchanger found in cone photoreceptors. Consistent with the loss of cones in *cep290* and *bbs2* mutants, *gucy2d*, *guca1c*, and *slc24a2*, were all downregulated in both *cep290* and *bbs2* mutants (Figure 3B).

Among the overrepresented molecules were several transcription factors that are associated with inflammatory and regenerative responses. Of the 37 identified transcription factors, 27 were upregulated and 9 were downregulated in both *cep290* and *bbs2* mutant retinas (Figure 3C). Three interferon regulatory factor (IRF) proteins are upregulated (*irf1b, irf3*, and *irf9*). The IRFs are key mediators of the interferons (IFNs) to boost the innate immune response (Tamura et al., 2008). IRF1 and IRF3 have been characterized as regulating expression of type I IFN genes in a number of cells (Schoggins et al., 2011). IRF9 associates with STAT1:STAT2 heterodimers to form the ISGF3 complex, which translocates to the nucleus to positively regulate transcription of type I and III interferon genes (Levy et al., 1989). Both *stat1a* and *stat3* were also upregulated, suggesting that retinal degeneration and inflammation triggers activation of the ISGF3-mediated interferon signaling. A third STAT family member, *stat3*, was also elevated in the mutants and its upregulation is essential for retinal regeneration in zebrafish (Nelson et al., 2012). The AP-1 transcription factor complex is a heterodimer composed of members of the Jun, Fos, and activating transcription factor (ATF) protein families. AP-1 induces expression of inflammatory markers and stimulates proliferation, differentiation, and apoptosis (Bejjani et al., 2019). AP-1 motifs were recently found to be a shared characteristic of the conserved regeneration-responsive enhancers (RREs) that activate regeneration in response to injury (Wang et al., 2020). Three ATF genes (*atf3, atf5a*, and *atf5b*), two Fos genes (*fosab* and *fosl1a*), and two Jun genes (*jun* and *junbb*) were upregulated, suggesting that upregulation of inflammatory markers was mediated by AP-1 in these mutants. The Myc genes *mych* and *myclb* were also upregulated, with a role in cell proliferation, cell cycle progression, and apoptosis. Inhibition of TGFβ signaling is necessary for Müller glia proliferation (Lenkowski et al., 2013) and the Tgif1 corepressor is rapidly upregulated in activated Müller glia after acute retinal injury to suppress TGFβ signaling (Lenkowski et al., 2013). Expression of *tgif1* is downregulated in both *cep290* and *bbs2*, consistent with the absence of Müller glia proliferation (Song et al., 2020; Fogerty et al., 2022). Expression of cone subtype-specific genes *foxq2* [blue cones (Ogawa et al., 2021)] and *sox6* [red cones (Ogawa and Corbo, 2021)] were also downregulated.

**Figure 4:**
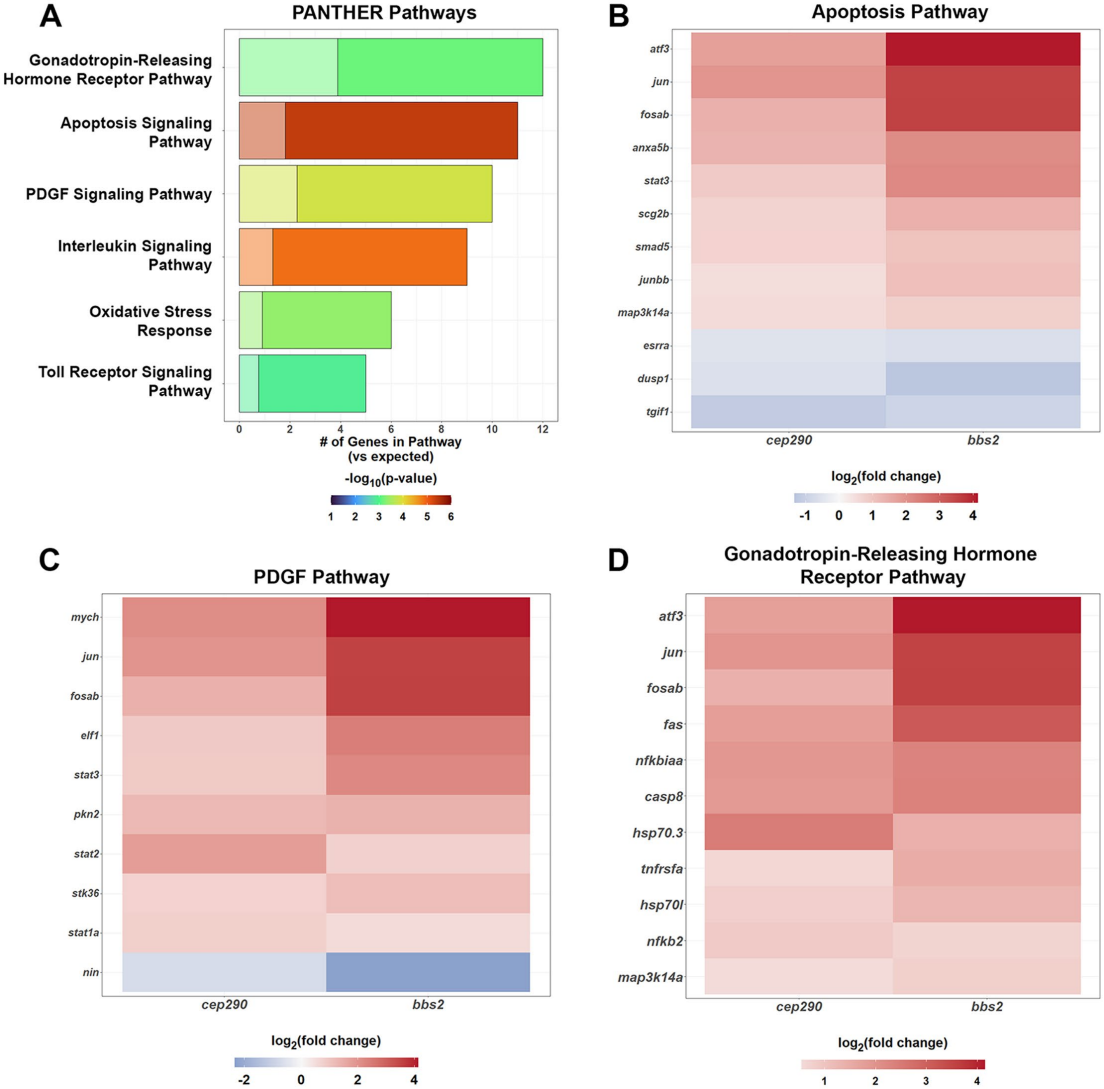
PANTHER Overrepresentation Analysis of the signaling pathways of the DEGs shared by *cep290* and *bbs2* mutants with a |log_2_ > 0.5| fold change in expression. **(A)** The DEGs shared between mutant retinas had an overabundance of genes in inflammatory and stress response signaling pathways. Genes of the apoptosis, PDGF, and gonadotropin-releasing hormone receptor pathway were also overrepresented. **(B)** The DEGs from the apoptosis signaling pathway were upregulated. **(C)** Genes of the platelet derived growth factor (PDGF) signaling pathway include the Stat transcription factors and were largely upregulated. The transcription factor *nin* was downregulated. **(D)** Genes of the gonadotropin-releasing hormone receptor pathway were largely upregulated and include *stat3*. Three transcription factors were downregulated.

Given the preponderance of signaling molecules and transcription factors among the top 50% of DEGs shared between *cep290* and *bbs2*, we used the PANTHER Pathway annotations to identify signaling pathways that may be activated. Upregulation of several inflammatory pathways were found in both these mutants, with genes involved in apoptosis, oxidative stress, Toll Receptor and Interleukin signaling pathways found to be more than 6-fold overrepresented among the top 50% of DEGs (Figure 4A). A number of upregulated transcription factors play active roles in these pathways. The transcription factors *jun* and *fosab* are found in apoptosis, the platelet-derived growth factor (PDGF) pathway and gonadotropin-releasing hormone receptor pathway (Figures 4B–D), suggesting a core set of transcription factors respond to chronic degeneration to activate several pathways. The transcription factors *atf3, jun, junbb, fosab*, and *stat3* are components of the apoptosis pathway (Figure 4B). In addition to the previously discussed *tgif1*, this pathway contains the downregulated transcription factor estrogen-related receptor alpha (*esrra*). Knockdown of *esrra* impairs the expression of *sox9* and *sox5* in zebrafish (Kim et al., 2015). *Sox9* is required for retinal differentiation and regulates the number of Müller glia and organization of photoreceptors (Yokoi et al., 2009). *Sox5* is expressed in resting Müller glia, is downregulated after injury, and is also regulated by *jun* and *junbb* (Hoang et al., 2020). These pathways may be important in the proliferation of rod precursors, which was noted in both *cep290* and *bbs2* models (Song et al., 2020; Fogerty et al., 2022). The sole downregulated gene in the PDGF pathway, Ninein (*nin*) is a centrosomal protein critical to the asymmetric cell division, and its absence resulted in the depletion of radial glia progenitors in mice (Wang et al., 2009). The transcription factors *atf3, jun*, and *fosab* are components of the gonadotropin-releasing hormone receptor pathway (Figure 4D). The upregulation of factors in the gonatotropin-releasing hormone receptor pathway was unexpected as this pathway regulates reproductive function in mammals. However, gonadotropin releasing hormone (GnRH) is expressed in the teleost retina where it plays a neuromodulatory role of dopaminergic activity in visual function (Zucker and Dowling, 1987; Grens et al., 2005).

**Figure 5:**
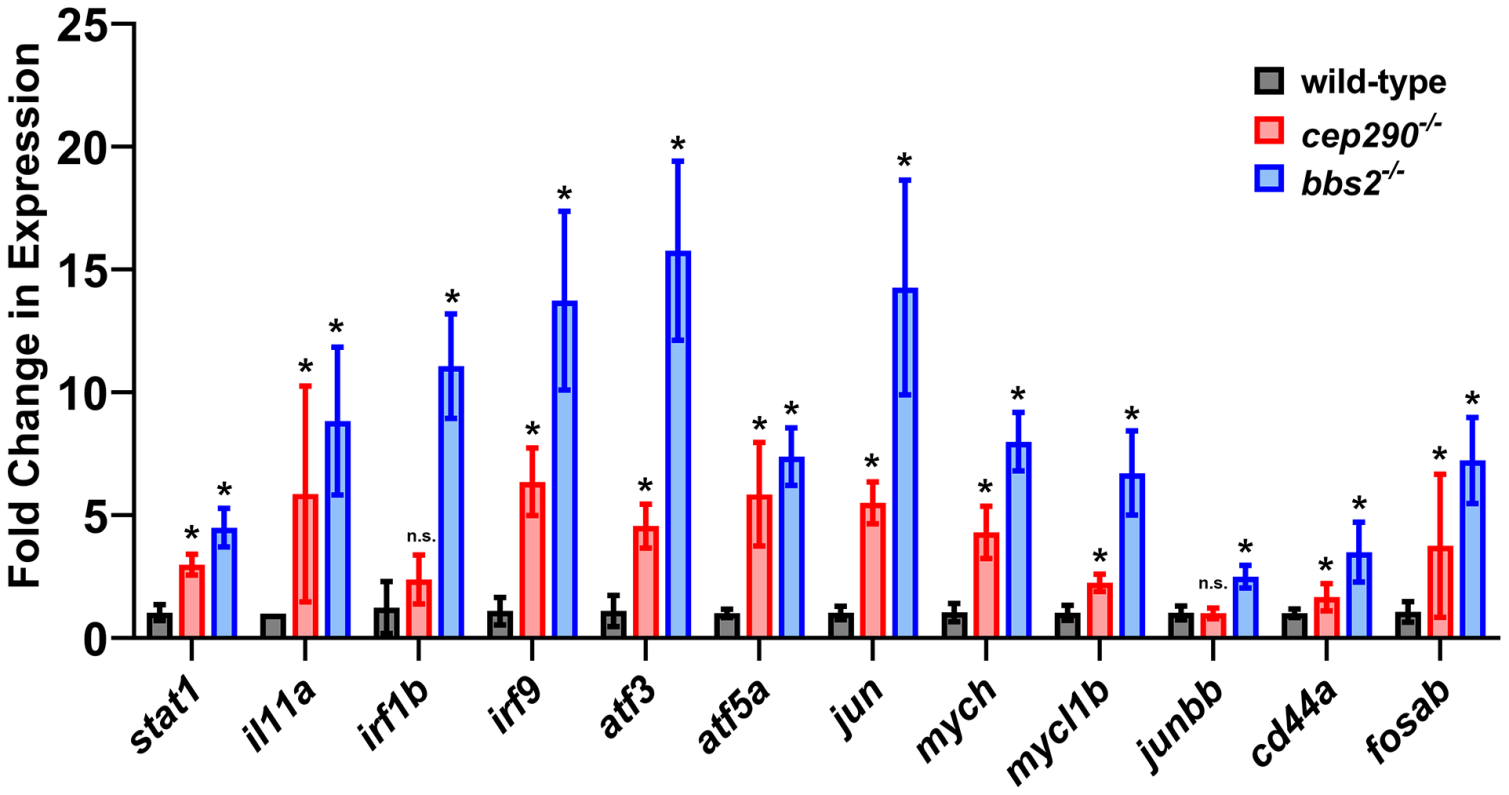
Fold change of gene expression in *cep290* and *bbs2* mutant retinas compared to wild-type animals. qRT-PCR of selected genes involved in inflammatory signaling and apoptosis processes in 6 mpf mutants. Gene expression was normalized to 18S reference gene expression and plotted relative to expression in wild-type siblings. Data are plotted as the mean ± S.D. Three technical replicates were conducted on each of four distinct biological samples of RNA. At least 2 retinas were pooled for each biological sample. *p < 0.05 by way of two-way Mann Whitney tests.

To validate our RNAseq results, we utilized quantitative real-time PCR (qRT-PCR) to examine the expression of several genes involved in inflammation and apoptosis (Figure 5). RNA was purified from retinas collected from 6 mpf *cep290* and *bbs2* mutants and from wild-type siblings. The RNA was reverse-transcribed into cDNA and used for qRT-PCR to assess gene expression for *stat1a, il-11a, irf1b, irf9, atf3, atf5a, jun, mych, mycl1b, junbb, cd44a*, and *fosab*. These genes were found to be overexpressed in both *cep290* and *bbs2* by RNAseq and are associated with multiple signaling pathways (Figure 4). In all but two cases, we found that these genes were expressed at statistically higher levels in *cep290* and *bbs2* mutants as compared to wild-type siblings. Only the expression of *irf1b* and *junbb* were not statistically different from wild-type controls. In the case of *irf1b*, the expression in *cep290* was 1.9-fold higher than control, which was similar to what was identified by RNAseq (1.45-fold), but the qRT-PCR failed to reach statistical significance (*p* = 0.11) due to high variability between samples. The expression of *junbb* in *cep290* mutants was only 0.5-fold higher by RNAseq. While the sensitivity of RNAseq data found this difference to be reproducible across the samples and statistically significant, we could not replicate this result using qRT-PCR. From these results, we concluded that the RNAseq data could be validated using qRT-PCR and that the RNAseq was often more sensitive and capable of detecting small, but reproducible changes in gene expression.

**Figure 6:**
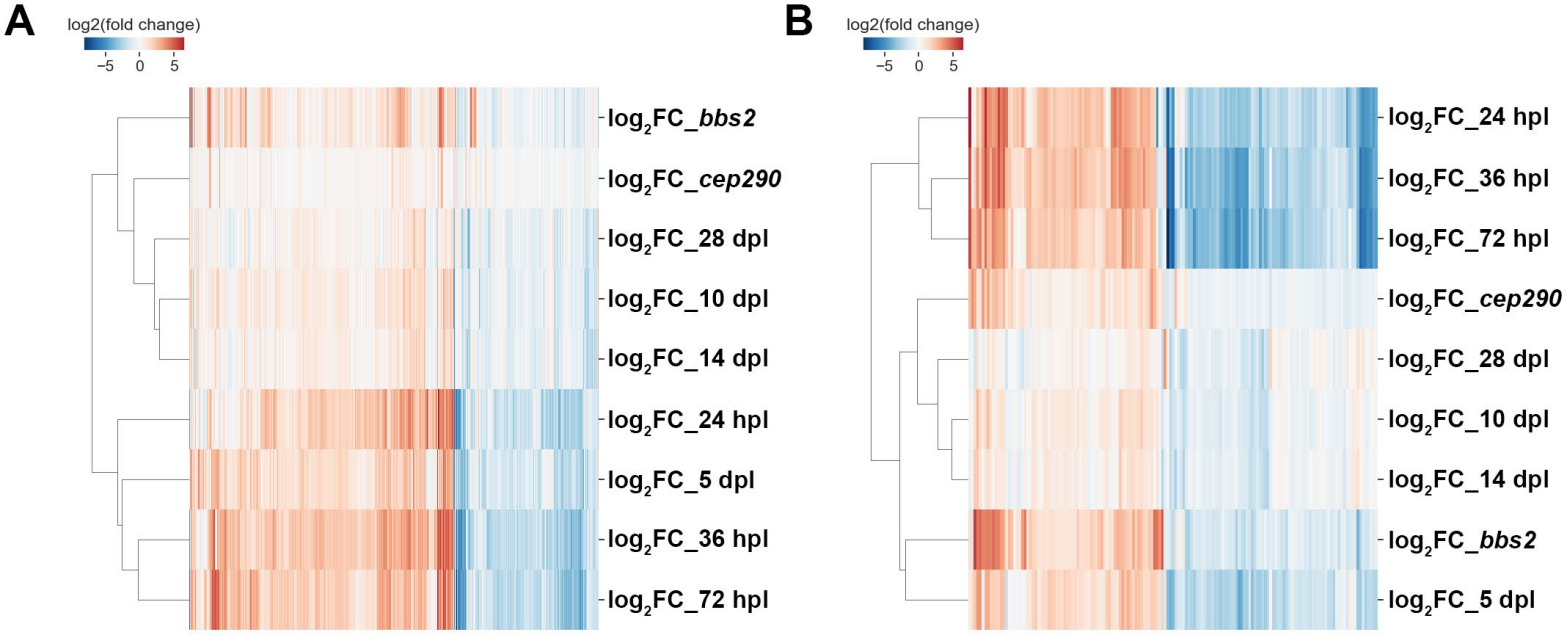
*cep290* and *bbs2* mutant gene expression as compared to a regeneration time series. **(A)** Differentially expressed genes across a 28-day retinal regeneration versus expression in *cep290* and *bbs2* versus wild-type. Expression in the mutants did not reach the levels seen between 24 h post-lesion and 5 days post-lesion and was most comparable to animals 10–28 days post-lesion. **(B)** 179 of the DEGs shared between *cep290* and *bbs2* were also differentially expressed between 24 and 72 h post-lesion, and the expression of these genes closely paralleled that of the actively regenerating animals.

As the *cep290* and *bbs2* mutants do not exhibit signs of regeneration but show evidence of inflammation, we asked how the gene expression patterns in *cep290* and *bbs2* mutants compared to the transcriptomes of wild-type animals at different stages of regeneration following acute light injury. A previous study provided detailed transcriptional profiling of wild-type retinas during early stages of regeneration at 24 h post lesion (hpl), 36 hpl, 72 hpl, and at ages of neuronal differentiation, such as 5 days post lesion (dpl) and 10 dpl, and at later points of functional recovery, such as 14 dpl and 28 dpl (Kramer et al., 2021). First, all individual DEGs from *cep290* and *bbs2* were hierarchically clustered with those DEGs identified in wild-type retinas over the course of a 28-day retinal regeneration (*n* = 1183). In aggregate, expression in the two mutants most closely resembles those of zebrafish at later stages of regeneration when proliferation has largely ceased and photoreceptors are differentiating (Figure 6A). When the analysis was restricted to the common genes differentially expressed in the two mutants (*n* = 179), the expression profiles most closely resembles the profiles from later stages of degeneration (Figure 6B). Genes of the Apoptosis and gonadotropin-releasing hormone receptor signaling pathways are also overrepresented among the 1,183 genes differentially expressed over this time series, suggesting that coordination of these two pathways is critical to the regeneration response.

## Discussion

This report provides a comprehensive transcriptomic analysis of *cep290* and *bbs2* mutant retinas at a time when photoreceptor degeneration is ongoing. Previous work had demonstrated that both *cep290* and *bbs2* mutants undergo slow retinal degeneration with increased retinal inflammation (Song et al., 2020; Fogerty et al., 2022). Zebrafish possess a remarkable ability to regenerate lost neurons in response to acute injury (Nagashima and Hitchcock, 2021) and it remains unclear why mutants with progressive, chronic degeneration, such as *cep290* and *bbs2*, fail to initiate a regenerative response. Transcriptional changes in response to acute damage to the zebrafish retina have been extensively documented (Kassen et al., 2007; Ramachandran et al., 2012; Sifuentes et al., 2016; Mitchell et al., 2019; Hoang et al., 2020; Kramer et al., 2021), while only a limited number of studies have examined transcriptional changes in retinal degeneration mutants (Morris et al., 2011; Saddala et al., 2020). The goals of this study were to identify transcriptional changes that were common between two models of retinal ciliopathies and to ascertain if those differences could provide insight on the reasons why these mutants do not exhibit signs of regeneration.

Analysis of the *cep290* and *bbs2* retinal transcriptomes found that both mutants share very similar gene expression profiles. Consistent with degeneration of photoreceptors, genes associated with phototransduction and light perception were downregulated. The enrichment of upregulated DEGs associated with protein synthesis and inflammatory signaling suggested that the zebrafish retina responds to photoreceptor loss by significantly increasing production of secreted molecules such as cytokines and other pro-inflammatory molecules. Inflammation plays an essential role in stimulating the initial reprogramming events that occur during retinal regeneration (Nagashima and Hitchcock, 2021). Prolonged exposure to inflammatory signaling, however, can also have negative consequence on regeneration (Silva et al., 2020). We previously reported that the reduction of microglia in *cep290; irf8* mutants lowered inflammation and prevented photoreceptor loss (Fogerty et al., 2022). Additional studies will be required to better understand the impacts of inflammation on photoreceptor loss and regeneration in models with chronic degeneration.

While gene expression in retinas from *cep290* and *bbs2* mutants differed significantly from gene expression in uninjured wild-type retinas, there was considerable similarity with the transcriptional profile observed in wild-type retinas between 5 and 28 days after acute light damage. At 5 days following injury, proliferating progenitor cells begin to differentiate into new photoreceptors (Raymond et al., 2006; Kramer et al., 2021). This corresponds to a significant shift in expression profiles from the early response to injury seen between 24 and 72 h post injury (Kramer et al., 2021). At later time points after injury, photoreceptors complete differentiation and markers of inflammation decrease. By 28 days following injury, photoreceptors appear to regenerate completely and appear similar to uninjured retinas (Vihtelic and Hyde, 2000; Kramer et al., 2021). Nevertheless, the transcriptome of retinas at the 28-day time point were notably different from uninjured retinas and share several similarities with that of the *cep290* and *bbs2* mutants. Thus, the transcriptional response to chronic degeneration in *cep290* and *bbs2* mutants was more similar to that of a morphologically and functionally regenerated retina than that of an uninjured zebrafish retina.

Bulk RNA-seq studies provide an insight into the gene expression of a tissue or cell population of interest and have both advantages and disadvantages. The approaches for conducting RNA-seq are widely accessible and data can be rapidly collected and analyzed to assess changes in gene expression. The data reflect changes in genes that are expressed at the time of tissue collection and often biased toward the most abundant transcripts in the samples, which may also reflect those transcripts found in the most abundant cell type in the tissue of interest. Here, we found a significant number of changes in expression of photoreceptor genes. This likely reflects both that photoreceptors are degenerating and that photoreceptors are among the most abundant cells in the retina. While genes expressed in microglia and Müller glia are contained in our data sets, the overall number of transcripts will be lower and this may limit the ability to detect significant changes in gene expression. As Müller glia are the source of neural progenitors that ultimately regenerate into photoreceptors, identifying changes in Müller glia gene expression will require the use of single cell RNA-seq (scRNA-seq) to provide higher resolution.

Overall, this report provides a foundation for subsequent comparative analyses with other zebrafish models of retinal degeneration, as well as mouse models of retinitis pigmentosa, to identify gene expression changes that contribute to photoreceptor death and for the discovery of pathways that regulate regeneration.

## Data availability statement

The datasets presented in this study can be found in online repositories. The names of the repositories and accession numbers can be found at: bbs2 RNAseq data – NCBI GEO, GSE223277. *cep290* RNAseq data – NCBI BioProject, PRJNA787536.

## Ethics statement

The animal study was reviewed and approved by the Cleveland Clinic Institutional Animal Care and Use Committee (IACUC).

## Author contributions

SG and BP conceived and designed the experiments, analyzed the data, and wrote and edited the manuscript and figures. SG, DP, and BP performed the experiments. All authors contributed to the article and approved the submitted version.

## Funding

This work was supported by NIH grants R01-EY017037 and R01-EY030574 and a Doris and Jules Stein Professorship Award from Research to Prevent Blindness BP. Additional support was provided by an NIH P30 core grant (P30-EY025585), a Foundation Fighting Blindness (FFB) Center Grant, and an Unrestricted Award from Research to Prevent Blindness to the Cole Eye Institute. The LRI Genomics Core is supported by an NIH P30 core grant (P30-CA043703). The funding agencies had no role in the design or execution of the research.

## Conflict of interest

The authors declare that the research was conducted in the absence of any commercial or financial relationships that could be construed as a potential conflict of interest.

## Publisher’s note

All claims expressed in this article are solely those of the authors and do not necessarily represent those of their affiliated organizations, or those of the publisher, the editors and the reviewers. Any product that may be evaluated in this article, or claim that may be made by its manufacturer, is not guaranteed or endorsed by the publisher.
